# Supplementary material for: Non-Ising and chiral ferroelectric domain walls revealed by nonlinear optical microscopy
Source: Nat Commun. 2017 Jun 8;8:15768. doi: 10.1038/ncomms15768 (PMC5472758; doi:10.1038/ncomms15768)
Supplement: Supplementary Information — Supplementary Figures, Supplementary Tables, Supplementary Notes, Supplementary Discussions and Supplementary References [file ncomms15768-s1.pdf]

Supplementary Note 1

Experiment

Detection of second-harmonic generation at domain walls

The spectral analysis of the optical signal detected at ferroelectric domain walls is obtained by shifting the emitted signal towards a spectrometer using a mirror (labeled M in Supplementary Fig. 1a). The spectrometry analysis of the intensity and wave length of the detected signal reveals a second-harmonic generation (SHG) effect at the domain walls (DWs).

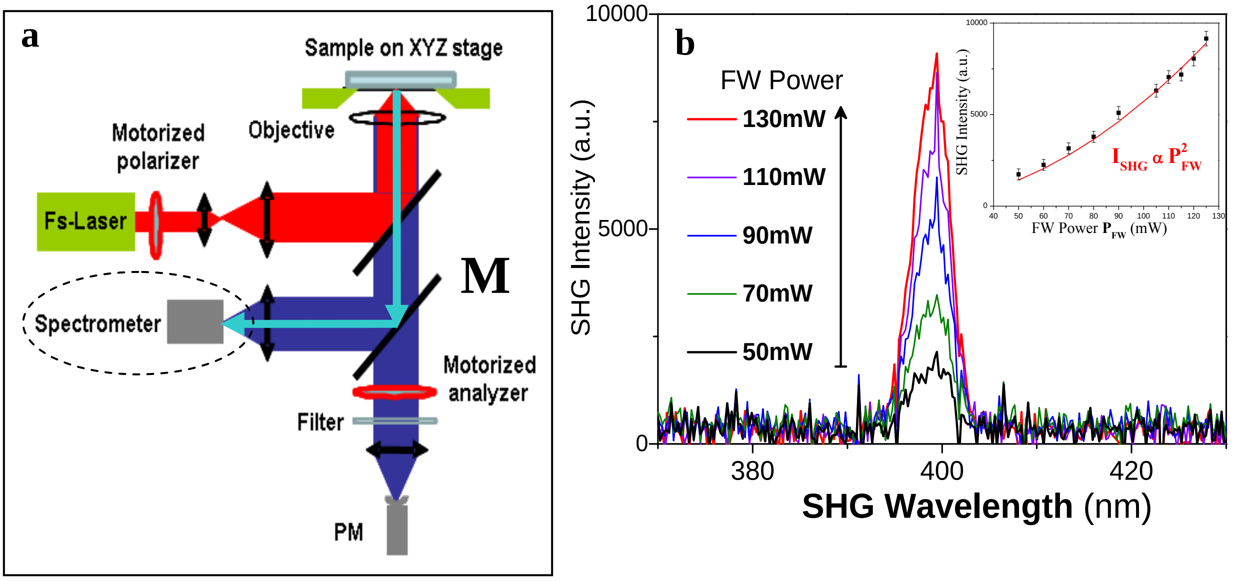

Supplementary Figure 1: Spectral analysis of the emitted signal. (**a**) Schematic representation of the experimental set up. The wavelength and the intensity of the emitted wave are measured using a spectrometer (dashed lines). (**b**) The wavelength of the emitted signal corresponds to the half of the fundamental wavelength and its intensity varies quadratically with the power of the incident signal.

Polarization dependence of the SHG: Local polar plots

A single SHG image is obtained by scanning the fundamental wave (FW) over a selected area of about 10 x 10 μm^2 and collecting the SHG signal at each *xy* scan step (typically 100 nm). The SHG intensity can be measured at different sets of the polarizer (FW polarization) and analyzer (SHG polarization) angles φ and α, as illustrated in Supplementary Figure 2.

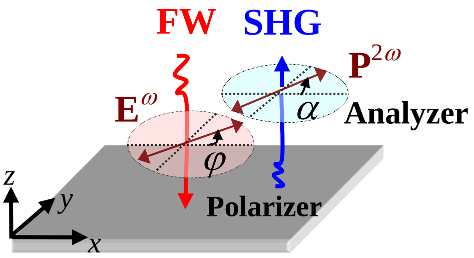

Supplementary Figure 2: **Schematic description of the polarimetry measurement procedure.** SHG polarimetry measurements are performed as a function of the polarization angle φ of the FW and the analyzer angle α of the SHG signal. The origin of both angles is taken with respect to the *x*-axis

Image series are recorded by varying the analyzer angle α in steps between 0 and 360° at a given FW polarization (*i.e.* φ fixed). Integration of the intensity over a selected area of the image sequence yields the local SHG variation as a function of the analyzer angle within the region of interest (ROI). To compare data from different regions, the obtained intensity value is normalized by the number of pixels of the selected ROI. The SHG intensities obtained in this way are therefore largely independent of the size of the selected area. In the case of thin film elements, the surface and the interface give rise to a background SHG signal. In order to obtain a better focus on the DWs, this background offset signal has been eliminated (the corresponding intensity was subtracted from the data). A typical polar plot of the SHG intensity for the case of horizontal and vertical DWs, respectively, is presented in Supplementary Figure 3.

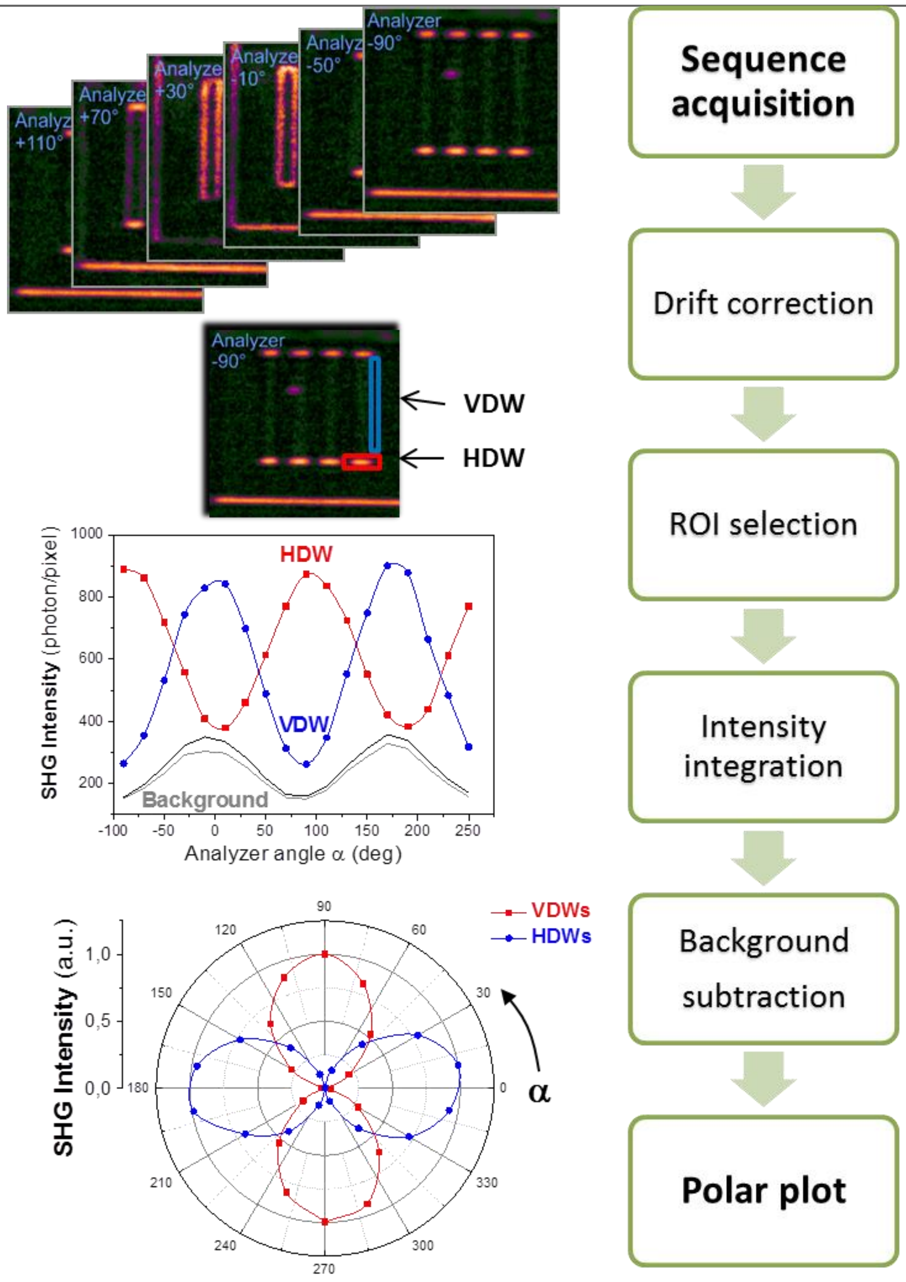

Supplementary Figure 3: Local SHG intensity analysis.
Polar plots at 180° domain walls in tetragonal $Pb(Zr,Ti)O_3$ thin films.

DW SHG is independent of the polarization orientation of the surrounding domains

As shown in Supplementary Figure 4, the SHG intensities measured in the case of 180° DWs are strictly independent from the sign of the polarization of the ferroelectric film within the surrounding domains, *i.e.*, the same result is obtained for domains with upward polarization written on a pre-poled downward-polarized background (Supplementary Fig. 4a) as for downward-polarized domains in an upward-polarized background (Supplementary Fig. 4d).

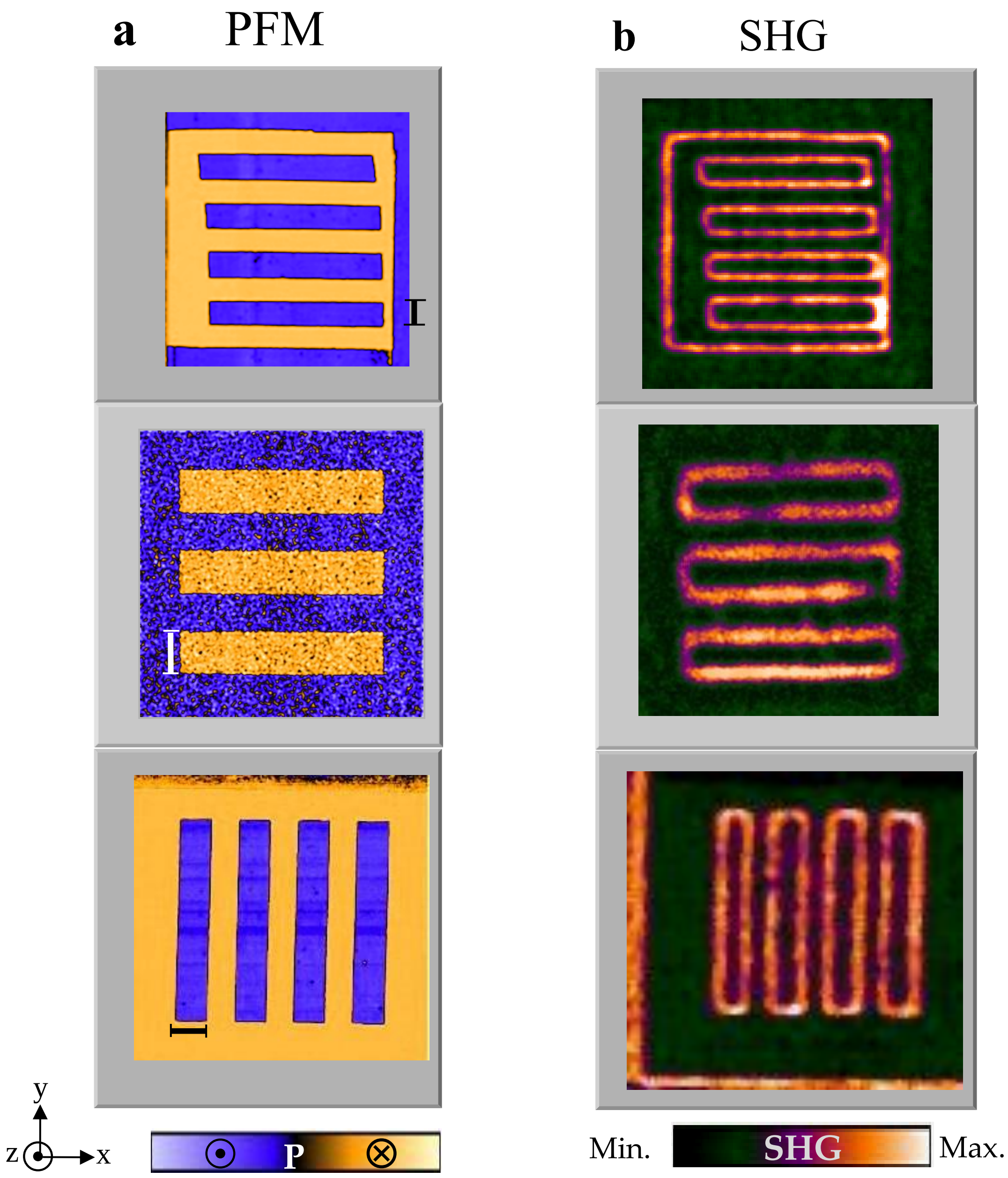

Supplementary Figure 4: Localized SHG at the DW regions in thin PZT films.
(**a**) Piezoresponse force microscopy images showing upward and downward polarized ferroelectric domains (c-domains); and (**b**) the corresponding SHG images.
The DWs' SHG emission is observed, irrespective of the sign of the polarization, *i.e.*, up-oriented domains on down-oriented background or *vice versa*. Also, the length of the walls along *x* or *y*-axis (long axis along *x* or *y*-axis) does not affect the SHG signal. The scale bar in all images corresponds to 2 µm

Analysis of the two-dimensional SHG profile at the DW regions

The typical intensity distribution across a series of 180° domain wall in PZT is shown in Supplementary figure 5a. Since the size of ferroelectric domain walls is of the order of a few unit cells, one can easily neglect the DW width compared to the size of the photon beam focus, itself of the order of 1µm. Their convolution means that analysis of the SHG profile cannot yield information about the local polarization structure within the domain walls, which is rather obtained through polarimetry analysis. The experimental data and simulation results are therefore interpreted on the basis of symmetry arguments. However, as shown in Supplementary Figure 5b, SHG profile analysis does provide an independent measurement of the instrumental resolution and FW characteristics as explained by Denev *et al.*[1].

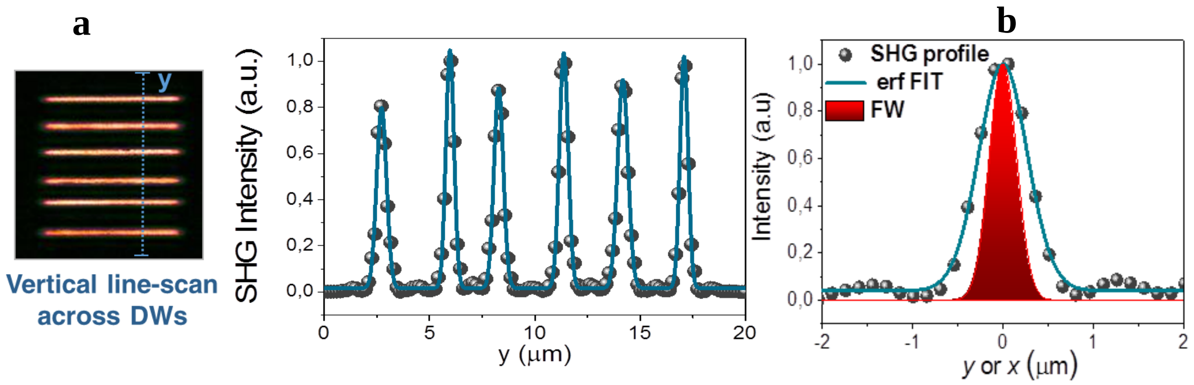

Supplementary Figure 5: SHG Profile across DWs.
(**a**) SHG Intensity profile measured across 180° DWs oriented along the x-axis (horizontal DWs), (**b**) extraction of the fundamental beam waist from the fit of the SHG profile following the method explained in Ref.1. The SHG profile is fitted with a Gaussian FW and a distribution of the 2D Gaussian profile while scanning the FW orthogonal to the DW is modeled by an error function (erf). This yields the fundamental beam waist ω_0 = 151 nm. Knowing the characteristics of the incident beam, the lateral resolution of the instrument is estimated by applying the Rayleigh criterion
(NA = 0.66): $R_{SHG} \sim 2.9 \times \omega_0 /\sqrt{2} = 309.6$ nm. This value is close to the theoretical diffraction limit $R_{diffraction} = 0.320 \times 800/(\sqrt{2} \times 0.66) = 274.3$ nm.
While useful information about the beam profile and the lateral resolution could be deduced from the intensity profile analysis, no information about the DW internal structure could be deduced.

Analysis of the three-dimensional profile of 180° DWs

A three-dimensional (3D) investigation of the DWs is conducted by means of 3D SHG microscopy[2,3] in nearly stoichiometric lithium tantalate bulk crystals in which periodic domains have been periodically poled (alternated c^+/c^- strip domains) by electric field throughout the 500 µm sample thickness. Due to the elongated shape of the laser beam in the *z*-direction (about 3 µm, as opposed to 1 µm along (*xy*)), the focal position is changed by moving the sample along the axial direction z in steps of about 10 µm. Due to the limited thickness of the PZT film (< 3µm), only $LiTaO_3$ crystals could be probed by this technique.

Supplementary Fig. 6(a,b) shows the polar plots recorded at different depths in the c-domains (background signal) and in the domain wall regions. In spite of a strong decrease of the absolute SHG intensity in the interior of the sample with respect to the surface due to a positive value of the SHG mismatch Δk_r (see Supplementary Ref 3), the local nonlinearity of the walls is clearly visible. We exploit the good signal-to-background ratio at the walls (SHG at c-domains is negligible) to construct 3D images of the wall regions as shown in Supplementary Fig. 6.

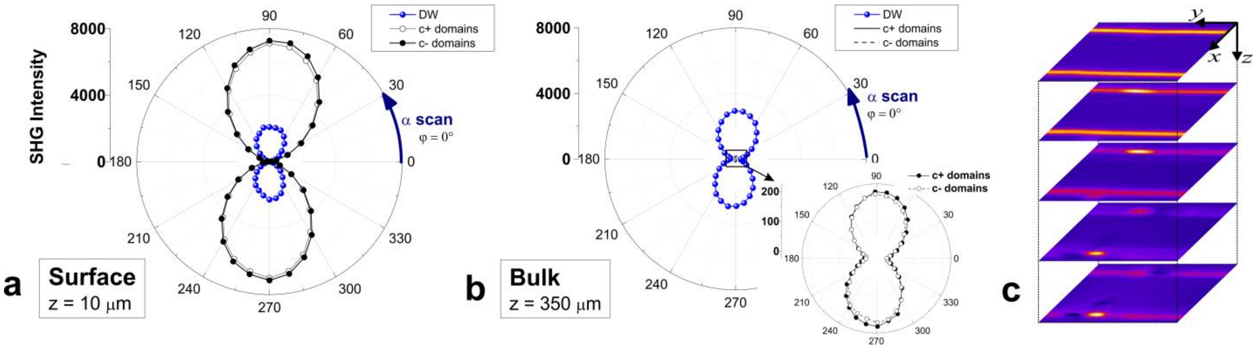

Supplementary Figure 6: 3D SHG mapping of 180° DWs in $LiTaO_3$. (**a**,**b**) Depth-dependent SHG polar plots and (**c**) 3D SHG Profile at 180° DWs in periodically poled nearly stoichiometric $LiTaO_3$.

Supplementary Note 2

Deriving the nonlinear optical susceptibility tensors

Polarization response to electromagnetic excitation

An electromagnetic wave traveling through a dielectric medium induces a charge separation, which creates local electrical dipoles, i.e. a polarization **P**, which is proportional to the electric field **E** of the incoming wave. While in a first approximation a linear dependence between the local field **E** and the induced polarization **P** can be assumed, nonlinear polarization effects become much more pronounced if the incident electric field is large (*e.g.* when an intense laser beam is used). In this case, the material response can be accounted for by expanding the polarization in a power series of the incident electric field:

$$P_i = \varepsilon_0 \sum_j \chi_{ij}^{(1)} E_j + \varepsilon_0 \sum_{j,k,\ldots} (D^{(2)} \chi_{ijk}^{(2)} E_j E_k + D^{(3)} \chi_{ijkl}^{(3)} E_j E_k E_l + \ldots) \qquad (1)$$

The first term in the Supplementary Eq. (1) represents the linear polarization and the higher order terms correspond to the nonlinear polarization response, where ε_0 is the vacuum permittivity, D is a degeneracy factor, χ is the macroscopic susceptibility of the material, superscripts (1), (2), (3) labels the order of the related tensor, and the indices i,j,k refer to laboratory coordinates. Second harmonic generation (SHG) is a nonlinear process which, microscopically, involves the coupling of two incident photons at frequency ω to produce a polarization oscillating with the double frequency $\mathbf{P}(2\omega)$. The leading term in the SHG frequency doubling process is the second order electric susceptibility $\chi^{(2)}$. This term contains the information related to ferroelectric ordering. The susceptibility tensor is usually replaced by a contracted d-tensor (Voigt notation): $2d_{ij} = \chi^{(2)}{}_{ikl}$ and in the case of SHG the degeneracy factor is $D^{(2)} = 1/2$.

The second order nonlinear polarization is given by:

$$\begin{pmatrix} P_x(2\omega) \\ P_y(2\omega) \\ P_z(2\omega) \end{pmatrix} = \varepsilon_0 \begin{pmatrix} d_{11} & d_{12} & d_{13} & d_{14} & d_{15} & d_{16} \\ d_{21} & d_{22} & d_{23} & d_{24} & d_{25} & d_{26} \\ d_{31} & d_{32} & d_{33} & d_{34} & d_{35} & d_{36} \end{pmatrix} \begin{pmatrix} E_x^2(\omega) \\ E_y^2(\omega) \\ E_z^2(\omega) \\ 2\,E_y(\omega)E_z(\omega) \\ 2\,E_x(\omega)E_z(\omega) \\ 2\,E_x(\omega)E_y(\omega) \end{pmatrix} \qquad (2)$$

The number of the non-vanishing d_{ij} elements is further reduced when considering the point group symmetry of the material according to Neumann's principle.

In our experiment, both the FW and the SHG emission propagate along the z-axis (normal incidence), and the detection is in the reflection geometry. The electric field of the FW components can then only lie within the (*xy*) plane ($E_x = E_0 \cos\varphi$, $E_y = E_0 \sin\varphi$, $E_z = 0$), where φ is the polarization angle of the FW measured from the *x*-axis. Thus:

$$\begin{pmatrix} P_x^{2\omega}(\varphi) \\ P_y^{2\omega}(\varphi) \\ P_z^{2\omega}(\varphi) \end{pmatrix} = \varepsilon_0 \begin{pmatrix} d_{11} & d_{12} & d_{13} & d_{14} & d_{15} & d_{16} \\ d_{21} & d_{22} & d_{23} & d_{24} & d_{25} & d_{26} \\ d_{31} & d_{32} & d_{33} & d_{34} & d_{35} & d_{36} \end{pmatrix} \begin{pmatrix} E_0^2 \cos^2\varphi \\ E_0^2 \sin^2\varphi \\ 0 \\ 0 \\ 0 \\ E_0^2 \sin 2\varphi \end{pmatrix} \qquad (3)$$

The polarization signal after traversing the linear output analyzer is obtained as a function of orientation of the analyzer angle α by using *Jones formalism* for a linear polarizer with a transmission angle α:

$$\begin{pmatrix} P_x^{2\omega}(\varphi,\alpha) \\ P_y^{2\omega}(\varphi,\alpha) \\ P_z^{2\omega}(\varphi,\alpha) \end{pmatrix} = \begin{pmatrix} \cos^2\alpha & \cos\alpha\sin\alpha & 0 \\ \cos\alpha\sin\alpha & \sin^2\alpha & 0 \\ 0 & 0 & 1 \end{pmatrix} \begin{pmatrix} P_x^{2\omega}(\varphi) \\ P_y^{2\omega}(\varphi) \\ P_z^{2\omega}(\varphi) \end{pmatrix} \qquad (4)$$

The variation of the SHG intensity as a function of the FW polarization angle φ and the output analyzer angle α is $I^{\mathrm{SHG}}(\varphi,\alpha) = |\mathbf{P}^{2\omega}(\varphi,\alpha)|^2$.

The rotation matrix transformations

In ferroelectric materials, the SHG intensity and its optical polarization dependence need to be considered with respect to the orientation of the ferroelectric polarization in the domain structure under consideration. The form of the d_{ij} tensor is isomorphic to the piezoelectric tensor at a given symmetry. Hence, the same transformation operations which hold in piezoelectrics also apply to the optical d_{ij} tensor. We thus use the piezoelectric constant rotation method to describe the 3D nonlinear susceptibility tensor in ferroelectric materials depending on the polarization orientation with the system coordinates[4]. Accordingly, knowing a reference susceptibility tensor d^0 defined in the crystallographic reference frame (*X*,*Y*,*Z*), any new tensor element corresponding to an arbitrary coordinate system (*X'*,*Y'*,*Z'*) with a particular ferroelectric polarization of the underlying material can be deduced from the general rotation matrix transformations as:

$$d_{ij}^{\mathrm{New}} = A_{ik} d_{kl}^{0} \alpha_{lj}^{-1}, \qquad (5)$$

where A is the rotation matrix (product of three individual rotations: ϕ, θ, ψ counterclockwise about Z, X' and Z'' (see Supplementary fig. 7).

$$A_{ij} = (a_{ij}) = \begin{pmatrix} \cos\psi & \sin\psi & 0 \\ -\sin\psi & \cos\psi & 0 \\ 0 & 0 & 1 \end{pmatrix} \begin{pmatrix} 1 & 0 & 0 \\ 0 & \cos\theta & \sin\theta \\ 0 & -\sin\theta & \cos\theta \end{pmatrix} \begin{pmatrix} \cos\phi & \sin\phi & 0 \\ -\sin\phi & \cos\phi & 0 \\ 0 & 0 & 1 \end{pmatrix}, \qquad (6)$$

and the elements of the transformation matrix α^{-1} are functions of the directional cosines:

$$\alpha_{ij}^{-1} = \begin{pmatrix} a_{11}^2 & a_{21}^2 & a_{31}^2 & 2a_{21}a_{31} & 2a_{31}a_{11} & 2a_{11}a_{21} \\ a_{12}^2 & a_{22}^2 & a_{32}^2 & 2a_{22}a_{32} & 2a_{32}a_{12} & 2a_{12}a_{22} \\ a_{13}^2 & a_{23}^2 & a_{33}^2 & 2a_{23}a_{33} & 2a_{33}a_{13} & 2a_{13}a_{23} \\ a_{12}a_{13} & a_{22}a_{23} & a_{32}a_{33} & (a_{22}a_{33}+a_{32}a_{23}) & (a_{12}a_{33}+a_{32}a_{13}) & (a_{22}a_{13}+a_{12}a_{23}) \\ a_{13}a_{11} & a_{23}a_{21} & a_{33}a_{31} & (a_{21}a_{33}+a_{31}a_{23}) & (a_{31}a_{13}+a_{11}a_{33}) & (a_{11}a_{23}+a_{21}a_{13}) \\ a_{11}a_{12} & a_{21}a_{22} & a_{31}a_{32} & (a_{21}a_{32}+a_{31}a_{22}) & (a_{31}a_{12}+a_{11}a_{32}) & (a_{11}a_{22}+a_{21}a_{12}) \end{pmatrix} \qquad (7)$$

As a starting point (d^0), we consider the tensor corresponding to the ideal Ising type DW structure, with the c-polarization axis along z. Any deviation from this idealized Ising case is characterized by the rotation angles ϕ, θ, ψ and the corresponding new tensor d^{New}. In the following, the crystallographic axes X, Y, Z coincide with the *x*, *y*, *z* laboratory coordinates and the identity transformation corresponds to $\phi = \theta = \psi = 0$.

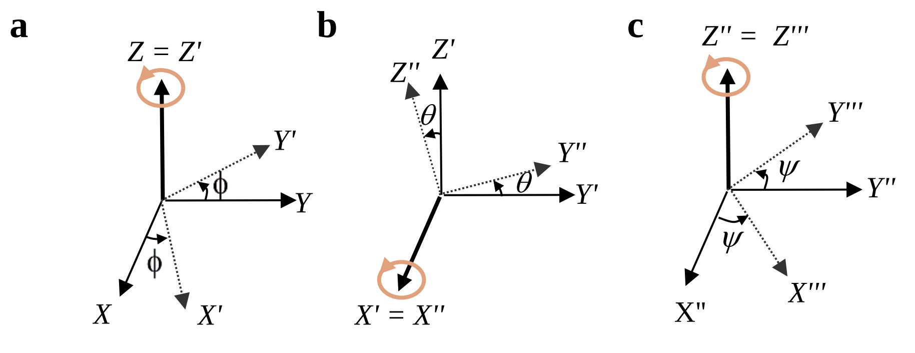

Supplementary Figure 7: **Rotation matrix transformation method.** Coordinate transformation (crystal to laboratory system) based on three counterclockwise angular rotations: (**a**) about *Z*, (**b**) about *X'* and, (**c**) about axis *Z''* as explained in Ref. 4.

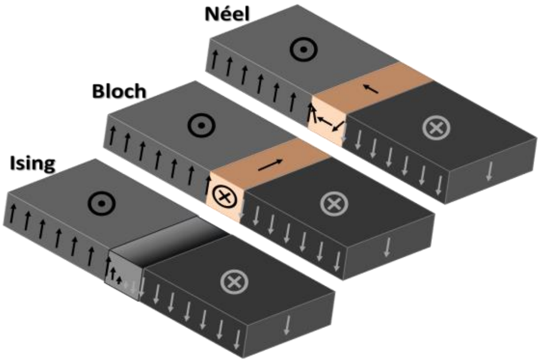

Supplementary Figure 8: **Schematic representation of the polarization at Néel, Bloch and Ising walls.** In this study, we consider an average planar polarization at the boundary region between opposite c-domains (light and dark gray regions). The in-plane polarization lays either perpendicular the walls in the case of Néel-type walls, or parallel to it in Bloch-type walls

The nonlinear optical susceptibility tensor in tetragonal Pb(Zr,Ti)O_3

The ideal Ising type DW (Supplementary Fig. 9) shows an abrupt transition between up- and down-oriented ferroelectric domains without any structural discontinuity (*i.e.* the same crystal structure is found on either side of the domain wall). At its center, such a domain wall region is furthermore centrosymmetric. Due to this symmetry and given the geometry of our measurement setup, it is expected that no SHG signal should be detected in this geometry for either the c-domains, or at the DWs in tetragonal PZT.

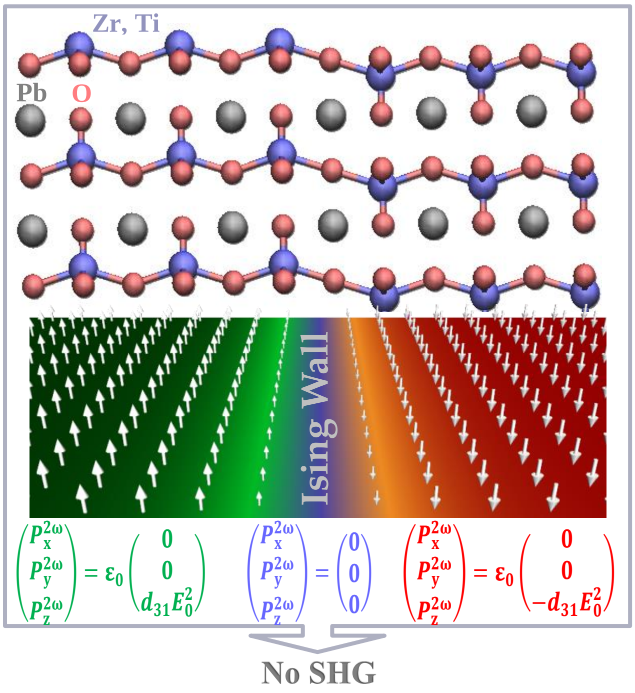

Supplementary Figure 9: **SHG at Ising-type walls and c-domains in tetragonal PZT.** The atomic structure of tetragonal PZT close to an ideal Ising-type 180° domain wall is shown in the upper part of the figure. Owing to the centrosymmetric character of the wall, no SHG is expected at the wall region. Besides, given the measurement geometry (normal incidence and back reflection detection), no SHG is also expected in the *xy*-plane from c-domains neither.

Supplementary Table 1: **Susceptibility tensors at 180° domain walls in tetragonal PZT.** The nonlinear optical susceptibility tensors are derived considering both Néel and Bloch-type configurations with horizontal (HDW), vertical (VDW) or oblique (ODW) orientation. The crystal axes coincide with the laboratory coordinate system. A and α are the rotation and transformation matrix given by the Supplementary equations (6) and (7), respectively.

Orientations	Types	(ϕ,θ,ψ) angles (°)	A_{ij}	α_{ij}	$d^{\text{New}} = A\, d^{o} \alpha^{-1}$
**Domains** // z-**axis**	c	(0,0,0)	NA	NA	$d^{0} = \begin{pmatrix} 0 & 0 & 0 & 0 & d_{15} & 0 \\ 0 & 0 & 0 & d_{15} & 0 & 0 \\ d_{31} & d_{31} & d_{33} & 0 & 0 & 0 \end{pmatrix}$
HDWs || x-axis	Néel	(0,90,0)	$\begin{pmatrix} 1 & 0 & 0 \\ 0 & 0 & 1 \\ 0 & -1 & 0 \end{pmatrix}$	$\begin{pmatrix} 1 & 0 & 0 & 0 & 0 & 0 \\ 0 & 0 & 1 & 0 & 0 & 0 \\ 0 & 1 & 0 & 0 & 0 & 0 \\ 0 & 0 & 0 & -1 & 0 & 0 \\ 0 & 0 & 0 & 0 & 0 & 1 \\ 0 & 0 & 0 & 0 & -1 & 0 \end{pmatrix}$	$\begin{pmatrix} 0 & 0 & 0 & 0 & 0 & d_{15} \\ d_{31} & d_{33} & d_{31} & 0 & 0 & 0 \\ 0 & 0 & 0 & d_{15} & 0 & 0 \end{pmatrix}$
HDWs || x-axis	Bloch	(0,90,90)	$\begin{pmatrix} 0 & 0 & 1 \\ -1 & 0 & 0 \\ 0 & -1 & 0 \end{pmatrix}$	$\begin{pmatrix} 0 & 1 & 0 & 0 & 0 & 0 \\ 0 & 0 & 1 & 0 & 0 & 0 \\ 1 & 0 & 0 & 0 & 0 & 0 \\ 0 & 0 & 0 & 0 & -1 & 0 \\ 0 & 0 & 0 & 0 & 0 & -1 \\ 0 & 0 & 0 & 1 & 0 & 0 \end{pmatrix}$	$\begin{pmatrix} d_{33} & d_{31} & d_{31} & 0 & 0 & 0 \\ 0 & 0 & 0 & 0 & 0 & d_{15} \\ 0 & 0 & 0 & 0 & d_{15} & 0 \end{pmatrix}$
VDWs || y-axis	Néel	(0,90,90)	$\begin{pmatrix} 0 & 0 & 1 \\ -1 & 0 & 0 \\ 0 & -1 & 0 \end{pmatrix}$	$\begin{pmatrix} 0 & 1 & 0 & 0 & 0 & 0 \\ 0 & 0 & 1 & 0 & 0 & 0 \\ 1 & 0 & 0 & 0 & 0 & 0 \\ 0 & 0 & 0 & 0 & -1 & 0 \\ 0 & 0 & 0 & 0 & 0 & -1 \\ 0 & 0 & 0 & 1 & 0 & 0 \end{pmatrix}$	$\begin{pmatrix} d_{33} & d_{31} & d_{31} & 0 & 0 & 0 \\ 0 & 0 & 0 & 0 & 0 & d_{15} \\ 0 & 0 & 0 & 0 & d_{15} & 0 \end{pmatrix}$
VDWs || y-axis	Bloch	(0,90,0)	$\begin{pmatrix} 1 & 0 & 0 \\ 0 & 0 & 1 \\ 0 & -1 & 0 \end{pmatrix}$	$\begin{pmatrix} 1 & 0 & 0 & 0 & 0 & 0 \\ 0 & 0 & 1 & 0 & 0 & 0 \\ 0 & 1 & 0 & 0 & 0 & 0 \\ 0 & 0 & 0 & -1 & 0 & 0 \\ 0 & 0 & 0 & 0 & 0 & 1 \\ 0 & 0 & 0 & 0 & -1 & 0 \end{pmatrix}$	$\begin{pmatrix} 0 & 0 & 0 & 0 & 0 & d_{15} \\ d_{31} & d_{33} & d_{31} & 0 & 0 & 0 \\ 0 & 0 & 0 & d_{15} & 0 & 0 \end{pmatrix}$
ODW1 +45° /x-axis	Néel	(0,90, 45)	$\begin{pmatrix} \sqrt{2}/2 & 0 & \sqrt{2}/2 \\ -\sqrt{2}/2 & 0 & \sqrt{2}/2 \\ 0 & -1 & 0 \end{pmatrix}$	$\begin{pmatrix} 1/2 & 1/2 & 0 & 0 & 0 & -1 \\ 0 & 0 & 1 & 0 & 0 & 0 \\ 1/2 & 1/2 & 0 & 0 & 0 & 1 \\ 0 & 0 & 0 & -\sqrt{2}/2 & -\sqrt{2}/2 & 0 \\ 1/2 & -1/2 & 0 & 0 & 0 & 0 \\ 0 & 0 & 0 & \sqrt{2}/2 & -\sqrt{2}/2 & 0 \end{pmatrix}$	$\sqrt{2}/4 \begin{pmatrix} (d_{15}+d_{31}+d_{33}) & -(d_{15}-d_{31}-d_{33}) & 2d_{31} & 0 & 0 & 2(d_{33}-d_{31}) \\ -(d_{15}-d_{31}-d_{33}) & (d_{15}+d_{31}+d_{33}) & 2d_{31} & 0 & 0 & 2(d_{33}-d_{31}) \\ 0 & 0 & 0 & 2d_{15} & 2d_{15} & 0 \end{pmatrix}$
ODW1 +45° /x-axis	Bloch	(0,90,-45)	$\begin{pmatrix} \sqrt{2}/2 & 0 & -\sqrt{2}/2 \\ \sqrt{2}/2 & 0 & \sqrt{2}/2 \\ 0 & -1 & 0 \end{pmatrix}$	$\begin{pmatrix} 1/2 & 1/2 & 0 & 0 & 0 & 1 \\ 0 & 0 & 1 & 0 & 0 & 0 \\ 1/2 & 1/2 & 0 & 0 & 0 & -1 \\ 0 & 0 & 0 & -\sqrt{2}/2 & \sqrt{2}/2 & 0 \\ -1/2 & 1/2 & 0 & 0 & 0 & 0 \\ 0 & 0 & 0 & -\sqrt{2}/2 & -\sqrt{2}/2 & 0 \end{pmatrix}$	$\sqrt{2}/4 \begin{pmatrix} -(d_{15}+d_{31}+d_{33}) & (d_{15}-d_{31}-d_{33}) & -2d_{31} & 0 & 0 & 2(d_{33}-d_{31}) \\ -(d_{15}-d_{31}-d_{33}) & (d_{15}+d_{31}+d_{33}) & 2d_{31} & 0 & 0 & -2(d_{33}-d_{31}) \\ 0 & 0 & 0 & 2d_{15} & -2d_{15} & 0 \end{pmatrix}$
ODW2 -45° /x-axis	Néel	(0,90,-45)	$\begin{pmatrix} \sqrt{2}/2 & 0 & -\sqrt{2}/2 \\ \sqrt{2}/2 & 0 & \sqrt{2}/2 \\ 0 & -1 & 0 \end{pmatrix}$	$\begin{pmatrix} 1/2 & 1/2 & 0 & 0 & 0 & 1 \\ 0 & 0 & 1 & 0 & 0 & 0 \\ 1/2 & 1/2 & 0 & 0 & 0 & -1 \\ 0 & 0 & 0 & -\sqrt{2}/2 & \sqrt{2}/2 & 0 \\ -1/2 & 1/2 & 0 & 0 & 0 & 0 \\ 0 & 0 & 0 & -\sqrt{2}/2 & -\sqrt{2}/2 & 0 \end{pmatrix}$	$\sqrt{2}/4 \begin{pmatrix} -(d_{15}+d_{31}+d_{33}) & (d_{15}-d_{31}-d_{33}) & -2d_{31} & 0 & 0 & 2(d_{33}-d_{31}) \\ -(d_{15}-d_{31}-d_{33}) & (d_{15}+d_{31}+d_{33}) & 2d_{31} & 0 & 0 & -2(d_{33}-d_{31}) \\ 0 & 0 & 0 & 2d_{15} & -2d_{15} & 0 \end{pmatrix}$
ODW2 -45° /x-axis	Bloch	(0,90, 45)	$\begin{pmatrix} \sqrt{2}/2 & 0 & \sqrt{2}/2 \\ -\sqrt{2}/2 & 0 & \sqrt{2}/2 \\ 0 & -1 & 0 \end{pmatrix}$	$\begin{pmatrix} 1/2 & 1/2 & 0 & 0 & 0 & -1 \\ 0 & 0 & 1 & 0 & 0 & 0 \\ 1/2 & 1/2 & 0 & 0 & 0 & 1 \\ 0 & 0 & 0 & -\sqrt{2}/2 & -\sqrt{2}/2 & 0 \\ 1/2 & -1/2 & 0 & 0 & 0 & 0 \\ 0 & 0 & 0 & \sqrt{2}/2 & -\sqrt{2}/2 & 0 \end{pmatrix}$	$\sqrt{2}/4 \begin{pmatrix} (d_{15}+d_{31}+d_{33}) & -(d_{15}-d_{31}-d_{33}) & 2d_{31} & 0 & 0 & 2(d_{33}-d_{31}) \\ -(d_{15}-d_{31}-d_{33}) & (d_{15}+d_{31}+d_{33}) & 2d_{31} & 0 & 0 & 2(d_{33}-d_{31}) \\ 0 & 0 & 0 & 2d_{15} & 2d_{15} & 0 \end{pmatrix}$

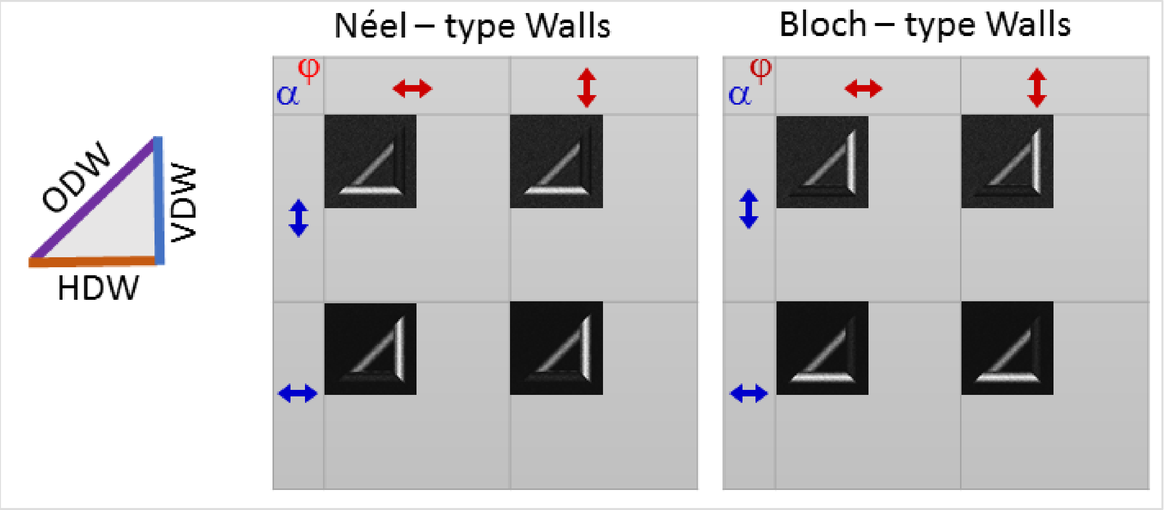

Supplementary Figure 10: 2D simulations of oblique walls in tetragonal PZT. The simulations are performed for a right angle triangle, assuming DWs with Néel-type Bloch-type internal structure. The variation of the SHG intensity at horizontal (HDW), vertical (VDW) and oblique (ODW) domain walls is displayed at different polarizer and analyzer angles (φ and α, respectively).

Supplementary Table 2: Numerical values of d_{ij} for barium titanate from the literature. Note that tetragonal barium titanate is known to exhibit comparable physical proprieties to tetragonal PZT.

		**Tetragonal $BaTiO_3$**		
		Theory	Experiment	Experiment
	Ref.	[5]	[6]	[7]
d_{ij} factors (pm/V)	d_{15}	-11.09	-17.7	-17.0
	d_{31}	-11.09	-18.8	-15.7
	d_{33}	-18.31	-7.1	-6.8

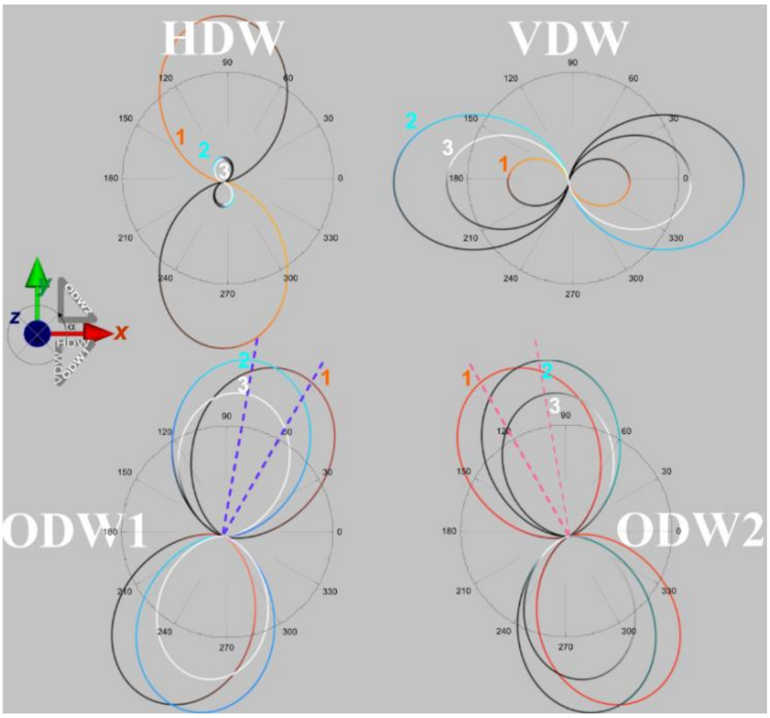

Supplementary Figure 11: **SHG variation with the nonlinear optical factors in tetragonal PZT**. Horizontal (HDW) and verticacal (VDW) walls are aligned along *x*-axis and y-axis, respectively, while ODW1 and ODW2 are at an angle of + (45 ± 5)° and - (45 ± 5)°, respectively, with respect to *x*-axis. Curves 1 (orange), 2 (cyan) and 3 (white) have been calculated using the d_{ij} factors after Ref [5-7] as summarized in Supplementary Table 2. The SHG intensity is presented in arbitrary units.

The nonlinear optical susceptibility tensor in trigonal $LiTaO_3$

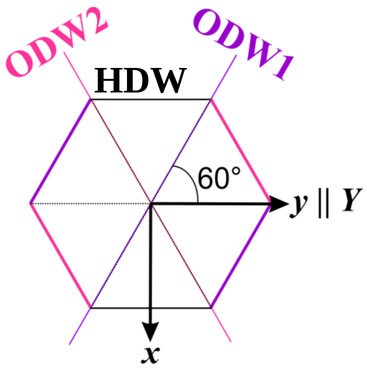

Supplementary Figure 12: **Schematic representation of c-domains in nearly stoichiometric LT.** The six walls of the hexagon are oriented along the *Y*-axis of the crystal.

In stoichiometric $LiTaO_3$ (LT), the DWs are aligned along the *Y*-axis of the trigonal crystal (see Supplementary Fig. 12).

For simplicity, we will consider that the crystal coordinates (*X*,*Y*,*Z*) coincides with the laboratory coordinates (*x*,*y*,*z*). Hence, the *Y*-walls defined below will be parallel to the y-axis in the laboratory coordinate system. As for the PZT film, the susceptibility tensor in Bloch and Néel like walls in LT is obtained by the rotation procedure described above (p. 7).

Supplementary Table 3: Susceptibility tensors at 180° domain walls in $LiTaO_3$. The nonlinear optical susceptibility tensors are derived for trigonal symmetry (point group 3*m*) considering both Néel and Bloch-type internal structure as well as different wall orientations**.** *A* and *α* are the rotation and transformation matrix given by equations (6) and (7), respectively.

Orientations	Types	(ϕ,θ,ψ) Angles (°)	A_{ij}	α_{ij}	$d^{New} = A\, d^{o} \alpha^{-1}$
**Domains // *z*-axis**	c	(0,0,0)	NA	NA	$d^{0} = \begin{pmatrix} 0 & 0 & 0 & 0 & d_{31} & -d_{22} \\ -d_{22} & d_{22} & 0 & d_{31} & 0 & 0 \\ d_{31} & d_{31} & d_{33} & 0 & 0 & 0 \end{pmatrix}$
**DWs || *y*-axis**	Néel	(0,90,90)	$\begin{pmatrix} 0 & 0 & 1 \\ -1 & 0 & 0 \\ 0 & -1 & 0 \end{pmatrix}$	$\begin{pmatrix} 0 & 1 & 0 & 0 & 0 & 0 \\ 0 & 0 & 1 & 0 & 0 & 0 \\ 1 & 0 & 0 & 0 & 0 & 0 \\ 0 & 0 & 0 & 0 & -1 & 0 \\ 0 & 0 & 0 & 0 & 0 & -1 \\ 0 & 0 & 0 & 1 & 0 & 0 \end{pmatrix}$	$\begin{pmatrix} d_{33} & d_{31} & d_{31} & 0 & 0 & 0 \\ 0 & 0 & 0 & d_{22} & 0 & d_{31} \\ 0 & d_{22} & -d_{22} & 0 & d_{31} & 0 \end{pmatrix}$
	Bloch	(0,90,0)	$\begin{pmatrix} 1 & 0 & 0 \\ 0 & 0 & 1 \\ 0 & -1 & 0 \end{pmatrix}$	$\begin{pmatrix} 1 & 0 & 0 & 0 & 0 & 0 \\ 0 & 0 & 1 & 0 & 0 & 0 \\ 0 & 1 & 0 & 0 & 0 & 0 \\ 0 & 0 & 0 & -1 & 0 & 0 \\ 0 & 0 & 0 & 0 & 0 & 1 \\ 0 & 0 & 0 & 0 & -1 & 0 \end{pmatrix}$	$\begin{pmatrix} 0 & 0 & 0 & 0 & d_{22} & d_{31} \\ d_{31} & d_{33} & d_{31} & 0 & 0 & 0 \\ d_{22} & 0 & -d_{22} & d_{31} & 0 & 0 \end{pmatrix}$
**ODW1 +60° /*y*-axis**	Néel	(0,90,-30)	$\begin{pmatrix} \frac{\sqrt{3}}{2} & 0 & \frac{-1}{2} \\ \frac{1}{2} & 0 & \frac{\sqrt{3}}{2} \\ 0 & -1 & 0 \end{pmatrix}$	$\begin{pmatrix} \frac{3}{4} & \frac{1}{4} & 0 & 0 & 0 & \frac{\sqrt{3}}{2} \\ 0 & 0 & 1 & 0 & 0 & 0 \\ \frac{1}{4} & \frac{3}{4} & 0 & 0 & 0 & \frac{-\sqrt{3}}{2} \\ 0 & 0 & 0 & \frac{-\sqrt{3}}{2} & \frac{1}{2} & 0 \\ \frac{-\sqrt{3}}{4} & \frac{\sqrt{3}}{4} & 0 & 0 & 0 & \frac{1}{2} \\ 0 & 0 & 0 & \frac{-1}{2} & \frac{-\sqrt{3}}{2} & 0 \end{pmatrix}$	$\begin{pmatrix} \frac{-1}{4}(3d_{31}+\frac{1}{2}d_{33}) & \frac{1}{4}(d_{31}-\frac{3}{2}d_{33}) & \frac{-1}{2}d_{31} & \frac{\sqrt{3}}{4}d_{22} & \frac{3}{4}d_{22} & \frac{\sqrt{3}}{4}d_{33} \\ \frac{\sqrt{3}}{4}(d_{31}+\frac{1}{2}d_{33}) & \frac{\sqrt{3}}{4}(d_{31}+\frac{3}{2}d_{33}) & \frac{\sqrt{3}}{2}d_{31} & \frac{1}{4}d_{22} & \frac{\sqrt{3}}{4}d_{22} & (d_{31}-\frac{3}{4}d_{33}) \\ \frac{3}{4}d_{22} & \frac{1}{4}d_{22} & -d_{22} & \frac{\sqrt{3}}{2}d_{31} & \frac{-1}{2}d_{31} & \frac{\sqrt{3}}{2}d_{22} \end{pmatrix}$
	Bloch	(0,90, 60)	$\begin{pmatrix} \frac{1}{2} & 0 & \frac{\sqrt{3}}{2} \\ \frac{-\sqrt{3}}{2} & 0 & \frac{1}{2} \\ 0 & -1 & 0 \end{pmatrix}$	$\begin{pmatrix} \frac{1}{4} & \frac{3}{4} & 0 & 0 & 0 & \frac{-\sqrt{3}}{2} \\ 0 & 0 & 1 & 0 & 0 & 0 \\ \frac{3}{4} & \frac{1}{4} & 0 & 0 & 0 & \frac{\sqrt{3}}{2} \\ 0 & 0 & 0 & \frac{-1}{2} & \frac{-\sqrt{3}}{2} & 0 \\ \frac{\sqrt{3}}{4} & \frac{-\sqrt{3}}{4} & 0 & 0 & 0 & \frac{-1}{2} \\ 0 & 0 & 0 & \frac{\sqrt{3}}{2} & \frac{-1}{2} & 0 \end{pmatrix}$	$\begin{pmatrix} \frac{\sqrt{3}}{4}(d_{31}+\frac{1}{2}d_{33}) & \frac{\sqrt{3}}{4}(d_{31}+\frac{1}{2}d_{33}) & \frac{\sqrt{3}}{2}d_{31} & \frac{-\sqrt{3}}{4}d_{22} & \frac{1}{4}d_{22} & -(d_{31}-\frac{3}{4}d_{33}) \\ \frac{-1}{4}(d_{31}-\frac{3}{2}d_{33}) & \frac{3}{4}(d_{31}+\frac{1}{2}d_{33}) & \frac{1}{2}d_{31} & \frac{3}{4}d_{22} & \frac{-\sqrt{3}}{4}d_{22} & \frac{\sqrt{3}}{4}d_{33} \\ \frac{1}{4}d_{22} & \frac{3}{4}d_{22} & -d_{22} & \frac{1}{2}d_{31} & \frac{\sqrt{3}}{2}d_{31} & \frac{-\sqrt{3}}{2}d_{22} \end{pmatrix}$
**ODW2 -60° /y-axis**	Néel	(0,90, 30)	$\begin{pmatrix} \frac{\sqrt{3}}{2} & 0 & \frac{1}{2} \\ \frac{-1}{2} & 0 & \frac{\sqrt{3}}{2} \\ 0 & -1 & 0 \end{pmatrix}$	$\begin{pmatrix} \frac{3}{4} & \frac{1}{4} & 0 & 0 & 0 & \frac{-\sqrt{3}}{2} \\ 0 & 0 & 1 & 0 & 0 & 0 \\ \frac{1}{4} & \frac{3}{4} & 0 & 0 & 0 & \frac{\sqrt{3}}{2} \\ 0 & 0 & 0 & \frac{-\sqrt{3}}{2} & \frac{-1}{2} & 0 \\ \frac{\sqrt{3}}{4} & \frac{-\sqrt{3}}{4} & 0 & 0 & 0 & \frac{1}{2} \\ 0 & 0 & 0 & \frac{1}{2} & \frac{-\sqrt{3}}{2} & 0 \end{pmatrix}$	$\begin{pmatrix} \frac{1}{4}(3d_{31}+\frac{1}{2}d_{33}) & -\frac{1}{4}(d_{31}-\frac{3}{2}d_{33}) & \frac{1}{2}d_{31} & \frac{-\sqrt{3}}{4}d_{22} & \frac{3}{4}d_{22} & \frac{\sqrt{3}}{4}d_{33} \\ \frac{\sqrt{3}}{4}(d_{31}+\frac{1}{2}d_{33}) & \frac{\sqrt{3}}{4}(d_{31}+\frac{3}{2}d_{33}) & \frac{\sqrt{3}}{2}d_{31} & \frac{1}{4}d_{22} & \frac{-\sqrt{3}}{4}d_{22} & -(d_{31}-\frac{3}{4}d_{33}) \\ \frac{3}{4}d_{22} & \frac{1}{4}d_{22} & -d_{22} & \frac{\sqrt{3}}{2}d_{31} & \frac{1}{2}d_{31} & \frac{-\sqrt{3}}{2}d_{22} \end{pmatrix}$
	Bloch	(0,90,-60)	$\begin{pmatrix} \frac{1}{2} & 0 & \frac{-\sqrt{3}}{2} \\ \frac{\sqrt{3}}{2} & 0 & \frac{1}{2} \\ 0 & -1 & 0 \end{pmatrix}$	$\begin{pmatrix} \frac{1}{4} & \frac{3}{4} & 0 & 0 & 0 & \frac{\sqrt{3}}{2} \\ 0 & 0 & 1 & 0 & 0 & 0 \\ \frac{3}{4} & \frac{1}{4} & 0 & 0 & 0 & \frac{-\sqrt{3}}{2} \\ 0 & 0 & 0 & \frac{-1}{2} & \frac{\sqrt{3}}{2} & 0 \\ \frac{-\sqrt{3}}{4} & \frac{\sqrt{3}}{4} & 0 & 0 & 0 & \frac{-1}{2} \\ 0 & 0 & 0 & \frac{-\sqrt{3}}{2} & \frac{-1}{2} & 0 \end{pmatrix}$	$\begin{pmatrix} \frac{-\sqrt{3}}{4}(d_{31}+\frac{3}{2}d_{33}) & \frac{-\sqrt{3}}{4}(d_{31}+\frac{1}{2}d_{33}) & \frac{-\sqrt{3}}{2}d_{31} & \frac{\sqrt{3}}{4}d_{22} & \frac{1}{4}d_{22} & -(d_{31}-\frac{3}{4}d_{33}) \\ \frac{-1}{4}(d_{31}+\frac{3}{2}d_{33}) & \frac{1}{4}(3d_{31}+\frac{1}{2}d_{33}) & \frac{1}{2}d_{31} & \frac{3}{4}d_{22} & \frac{\sqrt{3}}{4}d_{22} & \frac{-\sqrt{3}}{4}d_{33} \\ \frac{1}{4}d_{22} & \frac{3}{4}d_{22} & -d_{22} & d_{31} & -\frac{\sqrt{3}}{2}d_{31} & \frac{\sqrt{3}}{2}d_{22} \end{pmatrix}$

Supplementary Discussion

On Mixed Néel-Ising and Bloch-Ising characters

Supplementary Figure 13 shows 2D SHG simulations of DWs with mixed charter in tetragonal PZT. Dark regions are expected at the center of the walls, while the overall polarization dependence of the SHG remains analogous to the variation obtained in case of pure Bloch or Néel-type walls. The dark regions are related to the centrosymmetric Ising center with zero-polarization. These dark regions are not visible in our experiments probably because of the limited lateral resolution.

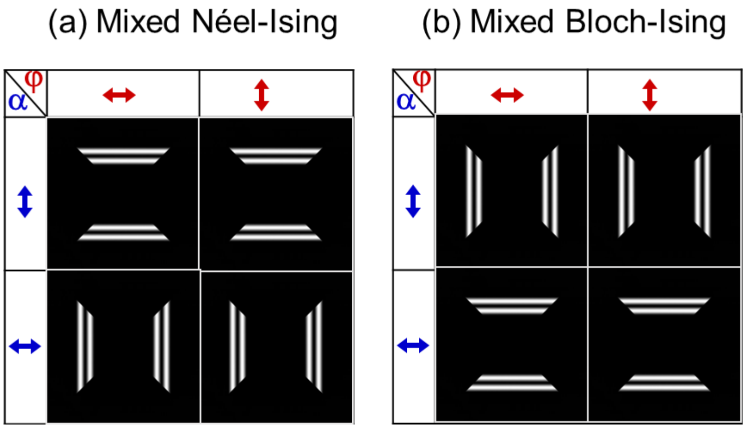

Supplementary Figure 13: 2D **Simulations of SHG at mixed domain walls in tetragonal PZT.** The (**a**) Néel-Ising and (**b**) Bloch-Ising mixed character of the walls are considered.

On the SHG detection in reflection geometry

In this study, the nondestructive reflection-type SHG geometry is employed. It is worth noting that the interpretation of the axial SHG signal requires specific precautions regarding possible phase matching effects affecting the SHG intensity as well as additional SHG contributions arising from symmetry breaking at the crystal surfaces.

Phase mismatch

In reflection geometry, the back-reflected forward SHG process is characterized by a wavevector mismatch $\Delta\mathbf{k}_r = \mathbf{k}(2\omega) - 2\mathbf{k}(\omega)$, where $\mathbf{k}(\omega)$ and $\mathbf{k}(2\omega)$ are the wavenumbers of second-harmonic and fundamental waves, respectively. Both are ordinarily polarized in our case. The very same configuration can also support backward SHG with a wavevector mismatch $\Delta\mathbf{k}_i = \mathbf{k}(2\omega) + 2\mathbf{k}(\omega)$, where $\mathbf{k}(2\omega)$ and $\mathbf{k}(\omega)$ are the same quantities defined above. In principle, both forward and backward signals contribute to the overall SHG intensity, although the former might be expected to be the dominant effects, in light of its larger coherence length (1.55 µm as opposed to a value of ~50 nm for backward SHG). Therefore, the phase mismatch affects the total SHG efficiency. However, when the electromagnetic wave propagates along the *z*-axis, both the second-harmonic and the fundamental waves are ordinary polarized (*i.e.*, polarized in the *xy*-plane). In this case, the values of $\Delta\mathbf{k}_r$, $\Delta\mathbf{k}_i$, and of the propagation distance (given by the crystal thickness) are constants. Their contribution affects the SHG intensity but it should not alter the shape of the SHG polar plots on which our conclusions are based. Note that this would not be the case if the experiments were done with waves propagating along *x* or *y*.

Surface SHG

Early studies have shown that SHG could originate at surfaces, even in centrosymmetric materials. This is due to the strong discontinuity of the electric field induced by the symmetry breaking at the surface. The specific nonlinear susceptibility tensors associated to surfaces may lead to a strongly different SHG response compared to that expected in the bulk of a given material. In particular, a specific nonlinearity (polar plot) and an increased intensity are expected at surfaces. These new tensor components at the surface – as compared to the bulk – may result in a deformation of the SHG polar plots. For this reason, in the article we discuss only the results obtained in the case when the fundamental laser beam is focused into the sample volume in the specific case of lithium tantalate. Moreover, since the surface SHG should be generated equally in the *xy*-plane, the surface contribution at the domain wall regions should be identical to that arising from the domains. Therefore, the specific SHG anisotropy at the domain wall regions (as compared to the domains) is unrelated to the uniform surface SHG. This background signal is subtracted in the case of PZT films, by following the method described in the Supplementary Figure 3. This contribution is insignificant in the case of $LiTaO_3$ bulk crystals, where the focus point is at 100 µm below the surface and the background signal is negligible.

Supplementary References

1. Denev, S. A., Lummen, T. T. A., Barnes, E., Kumar, A. & Gopalan, V. Probing Ferroelectrics Using Optical Second Harmonic Generation. *J. Am. Ceram. Soc.* **94,** 2699–2727 (2011).
2. Uesu, Y., Shibata, H., Suzuki, S. & Shimada, S. 3D Images of Inverted Domain Structure in $LiNbO_3$ using SHG Interference Microscope. *Ferroelectrics* **304,** 99–103 (2004).
3. Kaneshiro, J., Kawado, S., Yokota, H., Uesu, Y. & Fukui, T. Three-dimensional observations of polar domain structures using a confocal second-harmonic generation interference microscope. *J. Appl. Phys.* **104,** 54112 (2008).
4. Newnham, R. E. *Properties of Materials*. (Oxford University Press, 2005).
5. Hermet, P., Veithen, M. & Ghosez, P. Raman scattering intensities in $BaTiO_3$ and $PbTiO_3$ prototypical ferroelectrics from density functional theory. *J. Phys. Condens. Matter* **21,** 215901 (2009).
6. Zernike, F. & Midwinter, J. E. *Applied Nonlinear Optics: Basics and Applications*. (John Wiley & Sons Inc, 1973).
7. *Weber, M. J. (Ed.) Handbook of Laser Science & Techology, Vol. 3* (Chemical Rubber Company Press, 1986).
